# Factors Influencing the Long-Term Stability of Electronic Tongue and Application of Improved Drift Correction Methods

**DOI:** 10.3390/bios10070074

**Published:** 2020-07-07

**Authors:** Zoltan Kovacs, Dániel Szöllősi, John-Lewis Zinia Zaukuu, Zsanett Bodor, Flóra Vitális, Balkis Aouadi, Viktória Zsom-Muha, Zoltan Gillay

**Affiliations:** 1 Department of Physics and Control, Faculty of Food Science, Szent István University, Somlói út 14–16, H-1118 Budapest, Hungary; Zaukuu.John-Lewis.Zinia@hallgato.uni-szie.hu (J.-L.Z.Z.); Bodor.Zsanett@hallgato.uni-szie.hu (Z.B.); Vitalis.Flora@hallgato.uni-szie.hu (F.V.); Aouadi.Balkis@hallgato.uni-szie.hu (B.A.); Zsomne.Muha.Viktoria@etk.szie.hu (V.Z.-M.); Gillay.Zoltan@etk.szie.hu (Z.G.); 2 Institute of Pharmacology, Medical University of Vienna, 1090 Vienna, Austria; daniel.szoelloesi@meduniwien.ac.at

**Keywords:** drift correction, CHEMFET sensors, chemometrics, electrochemical, fingerprinting

## Abstract

Temperature, memory effect, and cross-contamination are suspected to contribute to drift in electronic tongue (e-tongue) sensors, therefore drift corrections are required. This paper aimed to assess the disturbing effects on the sensor signals during measurement with an Alpha Astree e-tongue and to develop drift correction techniques. Apple juice samples were measured at different temperatures. pH change of apple juice samples was measured to assess cross-contamination. Different sequential orders of model solutions and apple juice samples were applied to evaluate the memory effect. Model solutions corresponding to basic tastes and commercial apple juice samples were measured for six consecutive weeks to model drift of the sensor signals. Result showed that temperature, cross-contamination, and memory effect influenced the sensor signals. Three drift correction methods: additive drift correction based on all samples, additive drift correction based on reference samples, and multi sensor linear correction, were developed and compared to the component correction in literature through linear discriminant analysis (LDA). LDA analysis showed all the four methods were effective in reducing sensor drift in long-term measurements but the additive correction relative to the whole sample set gave the best results. The results could be explored for long-term measurements with the e-tongue.

## 1. Introduction

Generally, the human taste sensing system cannot be replaced by instruments. However, this sensory evaluation has many drawbacks like subjectivity and fatigue of the panel members and there are tasks where the human sensory evaluation is impossible for reasons ranging from safety to accuracy. Reasons such as these has led to a rising interest in instrumental alternatives with better advantages. The electronic tongue (e-tongue) and taste sensors were developed to satisfy such requirements. The principle and concept of these instruments are to measure samples with artificial sensors having cross-sensitivity and partial selectivity characteristics similar to the human tongue. The sensors are able to measure complex substances dissolved in liquids. The outcome of using such an instrument is a chemical pattern with characteristics for any sample. This is the so called “fingerprint” technology and is the main operating principle of artificial taste sensing devices.

E-tongue systems are typically made up of an autosampler, a sensor array, a signal acquisition device, and software for data processing [1,2], but the creation of this system generally requires expertise from different disciplines, e.g., sensory science, artificial intelligence, chemometric analysis [3]. Many types of chemical sensor array implementations have become widespread in e-tongue researches and practice. According to this, the literature distinguishes the electrochemical—potentiometric [4,5], voltammetric [6,7], amperometric [8], impedimetric [9], conductometric [10]—optical (florescence, absorbance, reflectance) [11], mass and biosensors [12] as the most common sensor types. In taste sensing measurements, the potentiometric e-tongue (“taste sensors”) are the most utilized but disadvantages such as strong temperature dependence and temporary or permanent solute sorption in sensor membranes can affect its sensitivity. However, and among the many advantages of the potentiometric measurement method used in the electronic tongue, it can be highlighted that its functional mechanism is the closest to the human component recognition process [13].

The investigations with potentiometric e-tongues are based on voltage measurement. There is no electric current between the sensors, thus, electrochemical reactions induced by the measurement do not proceed. Typically, Ag/AgCl electrodes are used as a reference point against the potentiometric working electrodes. The sensitivity of sensors is generally determined by the surface coat, which usually applies various lipid components embedded in the polymer membrane [12]. Contacts with the examined solution are formed depending on the selectivity of the membranes. Hence, the surface potentials develop and change accordingly, and this is detected by the device. Given that the obtained e-tongue data (sensor signals) are multicomponent, subsequent analyzes require the use of multivariate chemometric methods as tools for pattern recognition.

The first step in electronic tongue data analysis is visual inspection, which is used to determine what kind of pretreatment is required. Preprocessing is mainly performed in order to improve the signal-to-noise ratio, and to eliminate unnecessary data that can impair visualization.

From literature, there are publications dealing with instrumental taste sensing from the nineties. The number of the articles related to this topic increased continuously in the last three decades but there are only few publications that show the results of measurement comparisons performed in different times. This may be due to the issues of sensor instability associated with the electronic tongue instruments [14]. The instability of the chemical sensors is often referred to as sensor drift. It was also defined by Holmberg and Artursson [15] as follows: “a gradual change in any quantitative characteristic that is supposed to remain constant” but in general terms, it can refer to the inaccurate signal measurements of sensor-based instruments, mainly as a result of sensor deteriotion due to the evolution of the materials used in sensor development.

Drift is a major undesirable characteristic of sensor-based equipment, which could occur because of various known and unknown factors. Changes in the environmental conditions of the experiment, especially in temperature or unsatisfactory sensor cleaning, and even high variation in sample quality or concentration, are among the most known effects contributing to sensor drift. The response of the sensor depends on what it has recently been exposed to, because remnants of previous samples may be still present on the sensor surface [15]. For such reasons, measuring samples with highly different chemical composition and/or concentration may increase the probability of drift in the dataset; this phenomenon is often referred to as the memory effect. The transfer of a portion of the liquid sample on a macro scale due to unsatisfactory sensor cleaning is also a known contributor to changes in sensor signal, which is often called cross-contamination. Another consequence of the cross-contamination would be having some noise that has less to do with the multicomponent response and more to do with the interaction between contaminants and sensors through a series of adsorption/desorption reactions leading to limited performances [16]. These phenomena could be observed for all the different sensors of the e-tongue and could engender the inhibition of electrochemical reactions of interest, hence a loss of the electrodes activity [17]. From a technical and industrial perspective, drift problems hinder the long-term operation of all kinds of sensors [18,19,20]. The problem of drift associated stability is an even more important issue for the ion-selective field-effect transistor (ISFET) sensor-based electronic tongues. These instruments have high sensitivity, but are often associated with several disturbances [21]. Some studies have tried to deal with the issue of drifts in e-tongues, but this was not optimized and was not detailed.

According to most scientists in this field, e-tongues can be a useful instrument for quality monitoring and evaluation of various samples, but for this purpose, it is necessary to overcome the problem of the sensor drift [22]. Primarily, there are two major ways to decrease the drift: using mathematical corrections or improving the measurement system [23].

In improving the measurement system, practically controlling the temperature and using appropriate cleaning methods for the sensors have been recommended to decrease drift [24]. Regular polishing and calibration of voltammetric e-tongues with sandpaper and rinsing with distilled water have also been considered. These approaches however, lacked the time-efficiency needed for industrial applications and demand rigorous maintenance for long-term stability [25], but most importantly, they cannot be used in potentiometric sensors made up of highly sensitive membrane coating.

In addressing some of the occurring inaccuracies of such multidimensional data, several approaches have been adopted, but empirical mathematical logarithms have so far provided better outcomes [11]. Fundamental studies conducted by Davide et al. [26] aimed at elucidating the effect of gas sensor drifts from a mathematical stand point. Although their findings were effective, the approach fell short when dealing with dynamic variables such as concentration gradients. Contrarily, additive and multiplicative drift effects of MOSFET (metal oxide semiconductor field-effect transistor) and MOS (metal oxide semiconductor) gas sensors were successfully corrected using reference gas samples [27] and could also be useful in reducing e-tongue drift, but there are currently limited reports about their efficiency for long-term experiments (experiments exceeding 1–3 weeks of measurement). In an attempt to remove the gradual drift in the response of gas sensors and to avoid constant recalibrations, Artursson et al. [28] introduced a method known as the component correction (CC), inspired by orthogonal signal correction (OSC) from Wold et al. [29].

The proposed CC method helped in reducing the drift but its effectiveness was affected by occurring non-linearities as the tendency of the drift in the reference sample must be similar to that of the tested samples. Two data sets containing linear drift in multivariate spaces obtained by an e-tongue were nonetheless corrected by Holmin et al. [30] using the CC and additive correction method. The study, however, did not cover datasets measured in different days or weeks.

The most difficult expectation of e-tongue is a successful multi day comparison test. Thus, one of the most important goals for many real-life applications is to develop methods to achieve sensor signals stability even in long term. It is therefore necessary to explore and develop new and optimized drift correction methods for e-tongue applications.

Our research group has been working with an Alpha Astree (major manufacturers of electronic tongues) liquid and taste analyzer (usually referred as electronic tongue) on a wide range of applications, and we had several attempts and the opportunity to develop various stabilized drift correction techniques to improve the long-term stability of e-tongue measurements.

The aim of this paper was mainly to improve the reliability of the Alpha Astree e-tongue for long-term measurements by performing experiments to define the magnitudes of effect for temperature change between measurements, to prove that cross contamination can be a major factor in the observed signal changes beside other interfering factors, to confirm that the measuring order of the samples matters during measurements, and finally, to develop mathematical drift correction methods to eliminate or reduce the impact of drift on e-tongue results.

## 2. Materials and Methods

This paper focuses on three practically important major causes of drift: temperature change, cross contamination, and memory effect using apple juice and model solutions. The measurements were performed with the recent best measurement system and practices to decrease drift. Practical mathematical corrections were introduced and tested on datasets from the electronic tongue as described herein.

### 2.1. Instrumentation

An Alpha Astree liquid and taste analyzer (AplhaM.O.S., Toulouse, France), from now on referred as electronic tongue (e-tongue), was used in this study. The instrument was equipped with a 16-position autosampler and a sensor head consisting of a sensor array of seven ion selective field effect transistor (IFSET) based on potentiometric chemical sensors (ZZ, BA, BB, CA, GA, HA, JB) specialized for food applications and an Ag/AgCl reference electrode (Metrohm) and a stirrer. Each sensor is sensitive to chemical components in a dissolved liquid sample. This e-tongue operates on the principle that, the potential of the reference electrode remains constant so any measurable potential difference is related to the voltage change on the sensor membranes, representing the components present in the liquid sample [31]. The sensors are cross-selective and cross-sensitive to different chemical compounds. In such multisensory analyzes, each sample is characterized by the so-called fingerprint analysis method.

The instrument was setup according to the manufacturer’s instructions [31] before starting each measurement. This included a pre-conditioning with 0.01 M hydrochloric acid solution and the following two steps: a conditioning and a calibration step using the mixture of samples to be measured in equal portions. The aims of this preparation process were to condition the sensors to the analyzed samples and to reduce the possibility of the sensor signals being out of the measurement range during the experiment. The e-tongue was controlled by the software AlphaSoft, soft.ver. 12.3. 2.1.

### 2.2. Effect of Temperature Change on the Sensor Signals

Experiments were performed with apple juice samples at different sample temperatures of 5, 15, 25, and 35 °C. The apple juice samples were commercial products of 100% fruit juice content and were diluted with distilled water to obtain five different concentrations of 80, 85, 90, 95, and 100% (*v*/*v*).

A special thermostat vessel was designed and constructed to precisely determine the temperature dependence of the sensor signals of the e-tongue. The thermostat vessel was connected to an external water bath, providing the opportunity to keep the desired temperature of the samples constant during the experiment with a precision of ±0.2 °C using constant water circulation. Each sample was characterized by three replicates at each temperature level. The experiment was started with the acquisition of the cooled down samples at 5 °C and an additional blank sample was placed in the sequence to provide enough time for the samples to warm up to the next desire temperature and at the same time to prevent the sensor signals from being reconditioned. Therefore, the blank sample consisted of the mixture of samples to be measured in equal portions.

The apple juice samples measured at the different temperatures and different concentrations were placed in the autosampler according to increasing temperature, beginning with the sample of 5, 15, 25, and 35 °C in an increasing concentration level (80, 85, 90, 95, and 100%). Distilled water was placed between each sample in the autosampler as a cleaning solution for the sensors. The samples in the autosampler were in the following order: sample at 5 °C of 90 *v*/*v*%, then sample at 15 °C of 90 *v*/*v*%, sample at 25 °C of 80 *v*/*v*%, sample at 25 °C of 85 *v/v*%, sample at 25 °C of 90 *v/v*%, then sample at 25 °C of 95 *v/v*%, followed by the sample at 25 °C of 100 *v/v*%, and the sample at 35 °C of 90 *v/v*%. The temperature of the distilled water samples used as cleaning was also set to the desired temperature level.

### 2.3. Effect of Cross-Contamination

Each time, when the sensor head consisting of the seven sensors, reference electrode, and the stirrer moves from one sample holder to another, a certain amount of liquid is transferred from the previous sample to the next one through the surfaces of the above-mentioned elements. This amount of transferred liquid can alter the composition of the sample and influence the sensor signals. The e-tongue is suitable for measuring very small concentrations; thus, this cross contamination can also contribute to changed sensor signals during the measurement. As a counter measure, the manufacturer of the instrument recommends using distilled water in the sequence to clean the sensor head before each sample measurement. The cleaning parameters (speed, time, number of cleaning samples, and stirring rate of the stirrer) can also be chosen before the experiment and can be used as a means to optimize the cleaning of the sensor head, but the cleaning samples can still cause cross contamination due to sample adsorption on the surfaces of the element of the sensor head.

Although this confounding phenomenon ultimately affects the sensor signals, its direct impact cannot be singled out with monitoring of sensor signals alone because of other interfering parameters such as temperature etc., therefore, independent tools are needed. In this study, pH and the electrical conductance of the samples and their cleaning fluids were used to monitor this effect.

Commercial 100% apple juice samples and distilled water as cleaning fluid were used to test cross contamination. During the electronic tongue measurement, the pH of the apple juice samples and their cleaning fluids were measured before the 1st, 3rd, 5th, 7th, and 9th immersion of the sensor head in the respective beaker using the S40 SevenMulti™ pH meter. They were measured three times each and their averages were statistically analyzed.

### 2.4. Analysis of the Memory Effect

The sensors inherently have complex hysteresis (meaning on hysteresis the dependence of the state of a system on its history) type behavior which is not fully understood. The signal of the sensor changes over time, following an equilibrium after dipping into a given sample. In the fingerprinting approach, the sensor signal at the stage where the sensor is likely saturated with the target components of the liquid sample and reaches a stable level is used for the data evaluation. Hypothetically, this process can be disturbed by the memory effect caused by the previously measured samples by interfering with the sensitivity of the sensors. The memory effect is likely caused by components remaining chemically connected on the sensor membranes even after the cleaning process and can influence the equilibrated signals at the end of acquisition time [15]. Our hypothesis is that, this effect can cause significantly distinct changes during the repetition of the sample measurements, which can be considered as poor stabilization of the sensors. The memory effect can affect the discrimination of different samples by decreasing the difference of the signals between samples. This decreases the sensitivity of the system for the targeted samples. To avoid memory effect, the most obvious approach would be to completely clean the sensor, but it would need impractically a long time to reach the saturation, which is why the sensors need to be preconditioned in a sample close to the analyzed ones i.e., a mixture of the samples to be analyzed. In addition, lengthy periods of cleaning can enhance sensor deterioration.

It is assumed that model solutions, which are often applied as reference samples in drift correction methods, can alter the measurement of the target samples, therefore, the memory effect on the e-tongue sensor signals was analyzed using three different model solutions and apple juice samples. Citric acid, sodium chloride (NaCl), and mono sodium glutamate (MSG) solutions in 0.01 M concentration, corresponding to basic tastes, and 100% apple juice sample as a commercial product was used. The following experimental designs were applied to study the possible memory effect: In one arrangement, the samples were placed in the auto-sampler and analyzed in the following order (order a): citric acid, NaCl, MSG, and apple juice; in another arrangement, the reverse order (order b): citric acid, apple juice, MSG, NaCl; and finally (order c): citric acid, NaCl, MSG, apple juice sample order was applied. Between each dipping into the sample (measurement), the head was cleaned in distilled water. For both arrangements, nine complete measurement series of the samples were performed.

### 2.5. Experimental Setup and Development of New Drift Correction Methods

Experimental series with 100% commercial apple juice and 0.01 M concentration of citric acid, NaCl and MSG model solutions were executed from week 0 to week 5 to study the effect of drift on sensor signals of e-tongue and to determine the classification effectiveness of apple juice with drift corrections using these model solutions as reference points. The experiment of the apple juice and model solutions were performed once per week under controlled conditions as shown in Table 1.

Experimental conditions of temperature and cleaning solution were chosen and used throughout the six weeks experiment study on the effect of temperature and cross-contamination on the sensor signals. Distilled water was used as the cleaning solution and it was to clean the sensor head between the sample solutions in order to reduce cross-contamination.

For week 0 to week 3, the temperature of the samples was kept at constant 25 °C (±0.2 °C), with the same thermostat vessel used for the temperature experiment. The cleaning fluids were replaced with fresh ones between each repetition.

For week 4, the temperature of the samples was still kept at constant 25 °C (±0.2 °C), but the cleaning fluids were not replaced with fresh ones.

On week 5, the temperature of the samples was at around 23 °C, but was not controlled with the thermostat and the cleaning fluids were not replaced with fresh ones between the different repeats of the experiments.

The sequence of the samples and all the other measurement conditions were unchanged throughout the five weeks of the experiment except as clarified above. The sample preparation was controlled to avoid any unwanted change. Apple juice boxes with 100% fruit content from the same batch were used. The new apple juice box was opened, and 10 times dilution was freshly made every time right before the measurement. The model solutions were also freshly prepared with 0.01 M concentration using distilled water.

The instrument was pre-configured according to the manufacturer’s recommendation before each experiment by performing conditioning using 0.01 M HCl solution and distilled water, then a calibration using a mixture of the samples to be tested. There was no other measurement performed on the system during the six weeks of experiment. During conditioning, the analysis time (duration of sensors) in the 0.01 M HCl solution was 300 s but was 10 s in the cleaning solution. In total, this process took 56 min. During the calibration, the analysis time (duration of sensors) in the calibration sample was 120 s and the cleaning time was 10 s; calibration was repeated as many times as needed to calibrate the sensors. The measurement time for the tested samples was 120 and 10 s of cleaning. For the data analysis, the average of the last 10 s was taken and statistically analyzed. This time window was used to make sure the samples were well characterized by the equilibrium state of the sensors (the time at which the sensor signals are most stable). The sensors were rinsed with distilled water and left in open air to dry after each weekly experiment.

#### Description of the Drift Corrections Methods

For the purposes of this study, the signal obtained from the e-tongue using the manufacturer’s suggested protocol described above was referred to as raw data (non-drift corrected).

The appearing drift in the e-tongue-based analysis often makes the instrument unsuitable for long-term measurements, so three new drift correction methods were developed to test the long-term measurement of e-tongue using the data from the five-week experiment. The methods were developed in a simple but transparent way and were used to correct the drift occurring during the six weeks of experiments. For purposes of this study, they were referred to as additive correction relative to all samples, additive correction relative to reference samples, multi sensor linear correction (using reference samples), and the component correction based on principal component analysis (PCA) developed by Artursson et al. [28]. The calculation principles of the applied drift correction methods are detailed as follows.

(A) Additive correction relative to all samples

The idea of the additive correction relative to all samples is that the average sensor signals for all the repeats of a measurement without any disturbing variation needs to be constant. With any disturbance among the repeats, the change of the average sensor signal supposedly represents the caused drift. So, instead of using the original signal (*X*), it is more beneficial to subtract the average sensor signals for each sensor and for each repeat to get zero-centered values (*X′*) as shown in Equation (1):

(1) $${X^{\prime }}_{S}=X_{S}-\bar{X_{S}},$$

where *S* index stands for the sensors. Thus, each sensor and each repeat had a correction value. In this way, the “offset” type error between the measurements was corrected.

The key of this type of methods lies primarily in the simplicity of its calculation and secondarily that, there is no assumption about the behavior of the drift so, it can correct the effect of varying temperature or memory. This technique is appropriate in the cases where, measurements are performed with the same sample set and sequence.

(B) Additive correction relative to reference samples

This method was developed to overcome the limitation of the previous method that all the samples need to be the same in each measurement. In this method, the drift is checked based on the shift of the sensor signals of the reference samples. The rest of the samples are corrected based on the shift of the reference samples. The selected set model solutions as references were used to calculate the average sensor signal to subtract from the original signal for each sensor and for each repeat to get zero-centered values (*X′*) as shown in Equation (2):

(2) $${X^{\prime }}_{S}=X_{S}-\bar{X_{S}^{references}},$$

where *S* index stands for the sensors. Thus, each sensor and each repeat had a correction based on the selected references.

(C) Multi sensor linear correction (using reference samples)

The idea of the multi sensor linear correction is that the drift in the sensor signals between two measurements is not simply additive but also can be multiplicative. Thus, the mathematical relationship between sensor signals of the same samples on different days ($X^{1}$ and $X^{2}$) is linear. This relationship can be estimated by measuring reference samples along with the samples on both days (*Xref^1^* and *Xref^2^*). Using linear regression on reference signals *Xref^1^* and *Xref^2^* based on Equations (3) and (4) for each sensor:

(3) $$Xref_{S}^{1}=m\cdot Xref_{S}^{2}+b,$$

gives the slope (*m*), and the intercept (*b*) for the correction. The corrected signals (*X^2′^*) on the second day can be calculated from the original signals (*X^2^*) on the second day using the linear parameters (*m*, *b*):

(4) $${X_{S}^{2}}^{\prime }=m\cdot X_{S}^{2}+b.$$

where *S* index stands for the sensors. To determine the needed correction caused by drift, at least two reference samples are required to be measured. In this paper, we used three reference solutions as described above. The correction was applied for the measurements on different days but not for each repeat.

(D) Component Correction Based on PCA

According to Artursson et al. [28], one of the suggested drift correction methods was to remove the drift direction from the measurement matrix. The principle of this method is to determine the direction of the drift in the multi-dimensional sensor space by measuring a reference sample during the experiment. Basically, the principal component loadings of the reference sample ($X_{reference}$) are calculated during the drift correction process using PCA analysis. The PCA analysis finds the directions with the most variations. Thus, most likely, the highest variation in the reference sample measurements during the drift process is the direction of the drift so the first PCA loading (*p*) needs to be the direction of the drift. In this drift correction method, it is supposed that the drift direction in the reference sample is the same as the rest of the samples in the measurement. Using the first loading (*p*) to project the data set (X) to this direction will give the amount of change in the sensor signals in the direction of the drift (*t*), mathematically called the score of the first PCA component:

(5) t=X⋅p,

with this PCA score (*t*), the change of the signals in the drift direction, form the perpendicular hyper plane, as a matrix of vectors ($X_{drift}$) can be calculated:

(6) $$X_{drift}=t\cdot p^{T},$$

and $X_{drift}$ needs to be subtracted from the original matrix of the sensor signals to get the corrected sensor signal matrix:

(7) $$X_{corrected}=X-X\mathrm{drift},$$

for the whole data set.

**Figure 1:**
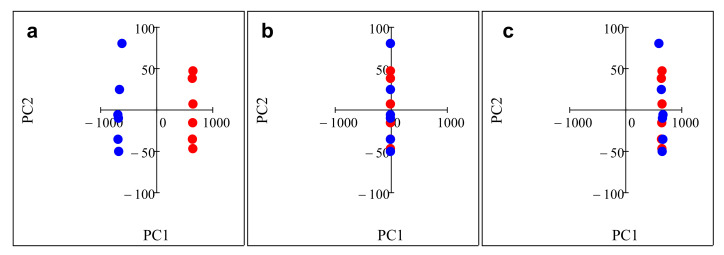
Principal component analysis plots demonstrating component correction for citric acid week 0 (red points) and week 1 (blue points). (**a**) original data and (**b**) component correction and (**c**) modified component (correction subtracting twice the drift from week 1).

This method is often effective in correcting drift, but there are disadvantages of this method. The corrected sensor signals do not contain any information in the direction of the drift and one sample can be used as reference. Furthermore, our drift process covered six weeks. When we calculated separate drift directions for each week relative to week 0, the corrected week 0 data were different and could not be used in discriminant analysis for the whole process. For this reason, first, we determined the correction using only week 0 and week 5. Then, tried to separate correction for each week but instead of correcting both week 0 and later weeks, we corrected only the later ones ($X^{2}$), subtracting twice the drift:

(8) $$X_{corr}^{2}=X^{2}-2\cdot X^{2}\mathrm{drift},$$

with this correction, instead of losing the information in the drift direction, we transformed the data of later weeks onto the data of week 0. Component correction is very sensitive for outliers, which is why it is important to carefully remove them. We determined and removed two outliers of apple juice and citric acid on week 2. Chemically, citric acid is the closest in composition to apple juice, which is why we used it as the reference to calculate the drift direction. The transformation is demonstrated in Figure 1.

### 2.6. Applied Statistical Methods and Software

As a first step in the data evaluation, principal component analysis (PCA) was performed. Based on the PCA results, outlier detection and data pretreatment were implemented. Based on this, the first three repetitions of the measurements proved to be outliers. This was most likely due to the fact that e-tongue sensors were not properly conditioned during that time of measurement, and therefore, were omitted before the further evaluation.

The results of the temperature experiments performed on apple juice samples of different concentrations were evaluated by linear discriminant analysis (LDA). In the case of the temperature effect measurement, the LDA models were built to discriminate between the different concentrations of apple juices at different temperatures. In addition to LDA, Euclidean multidimensional distances were calculated between the group centers of apple juice samples for the different concentrations and temperatures in the seven-dimensional space (from the seven e-tongue sensors), to compare the effects of temperature and concentration on sensor signals.

The pH results of the cross-contamination study were evaluated by calculating univariate linear regressions between the number of repeats and pH of the tested apple juice samples.

PCA was used to analyze the data obtained to investigate the memory effect. Three different PCA models were built for the three different experiments to visualize patterns between the tested apple juice and model solutions. The deviations of the different groups on the PCA score plots were compared.

The effectiveness of the applied drift correction methods in comparison to the non-corrected dataset was evaluated by LDA. Classification models were built using the data from week 0 to discriminate between the four solutions (citric acid, NaCl, MSG, and apple juice). These models were used to predict the data of experiments from week 1 to week 5, separately for each of the developed drift correction methods (i.e., non-drift corrected, additive correction relative to all samples, additive correction relative to reference samples, and multi sensor linear correction). The results of the three drift correction methods were compared to non-drift corrected ones by evaluating the relative pattern and deviation of the different groups of the model solutions and apple juice samples on the LDA score plots and by the change of the correct classification rate from one week to the next. In addition to the LDA evaluation, multidimensional Euclidean distances were separately calculated for each method between the group centers of the apple juice sample measured on week 0 and all the respective weeks of experiments separately in the three-dimensional LDA space normalized by the distance of the center of MSG and citric acid measured on week zero.

All the LDA models were validated by threefold cross-validation [32]. The predictive significance of each LDA model was tested by splitting the data into two groups: the training set and validation set. The training set consisted of two-thirds of the data and the validation set consisted of the remaining one-third of the data of each sample. The data splitting was repeated three times by substituting the one-third of the data in both the calibration and validation sets.

The additive correction relative to all samples and additive correction relative to reference samples were executed in R-project ver. 3.6.3. The multi sensor linear correction was done in MathCad ver. 14.0 (PTC) mathematical. The programs were used to complete mathematical transformation after raw data input, which was followed by the extraction of the corrected data matrixes. The LDA models were built and validated in Statistica ver. 9.1. with general discriminant analysis module [33].

## 3. Results

### 3.1. Effect of Temperature on the Sensor Signals

**Figure 2:**
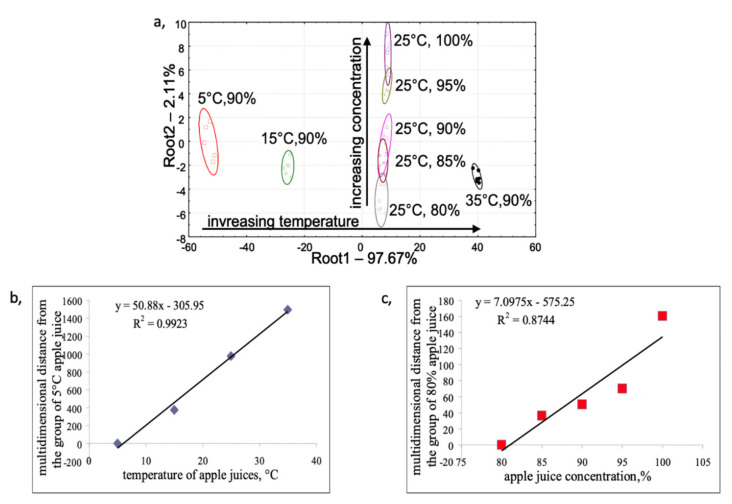
(**a**) Discriminant analysis score plot of the different concentrations of apple juice at different temperatures and (**b**) the Euclidian multidimensional distance between the groups of apple juices with different concentrations and (**c**) with different temperatures with the fitted regression models.

Figure 2, shows the result of discriminant analysis for the different concentrations of apple juice samples at different temperatures (a) and the Euclidian multidimensional distance between the groups of apple juices with different concentrations (b) and with different temperatures (c). The groups of lowest temperature (5 °C) and apple juice concentration (80%) were considered as the reference samples for the distance calculations, respectively.

### 3.2. Effect of Cross Contamination

**Figure 3:**
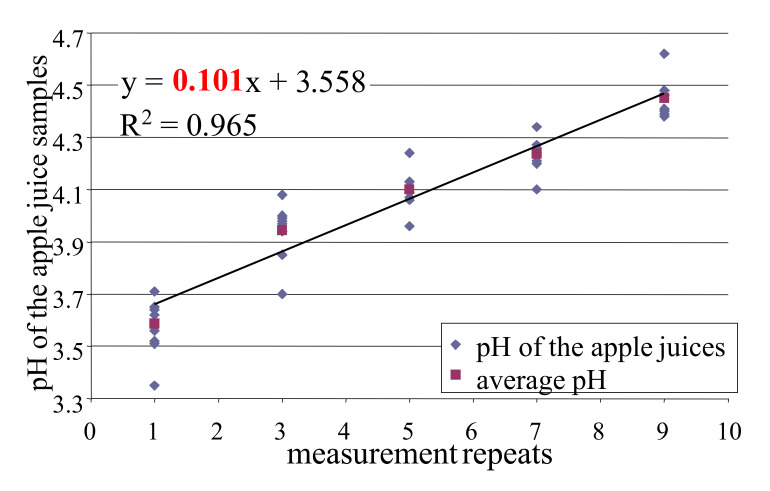
The increase of pH of apple juice samples versus electronic tongue measurement repeats.

The results in the pH change of apple juice samples in the function of the measurement repeats of the electronic tongue tests are shown on Figure 3. The regression model showed close correlation between the pH and number of measurements with a coefficient of determination (R^2^) of 0.965. The slope of the fitted model implies an average of about 0.1 pH increase by one-time transfer of distilled water from the cleaning fluid to the apple juice samples.

### 3.3. Analysis of Memory Effect

**Figure 4:**
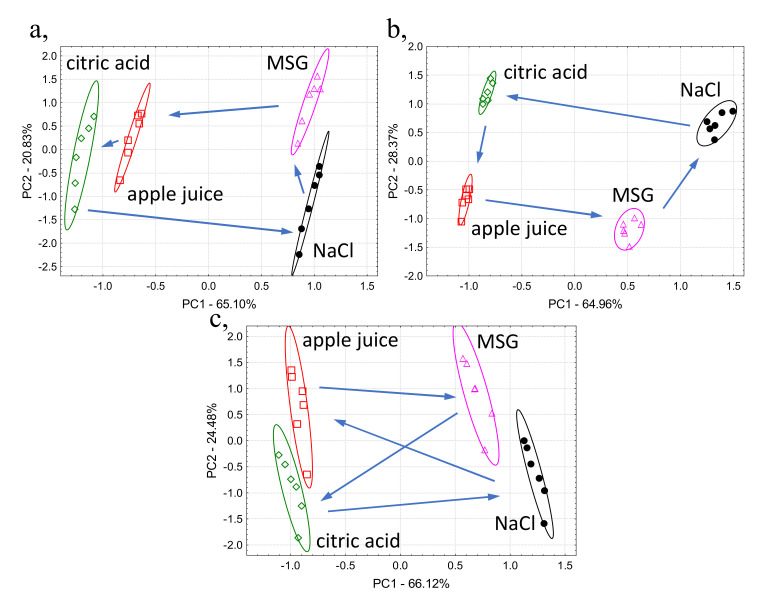
Principle component analysis score plots of the electronic tongue results of apple juice and model solution samples measured in three different sample orders: (**a**) citric acid NaCl, MSG, apple juice and: (**b**) reverse sample order: citric acid, apple juice, MSG, NaCl and: (**c**) citric acid, NaCl, apple juice, MSG.

Figure 4 shows the results of principle component analysis for apple juice and model solutions measured in different sample orders for the evaluation of memory effect Figure 4a presents the PCA results of the experiment where the sample order was: citric acid, NaCl, MSG, apple juice; and in Figure 4b, the one when the reverse sample order was: citric acid, apple juice, MSG, NaCl; and in Figure 4c, the sample order was applied: citric acid, NaCl, apple juice, MSG.

### 3.4. Development and Comparison of New Drift Correction Methods

#### 3.4.1. Raw Data (Non-Drift Corrected)

**Figure 5:**
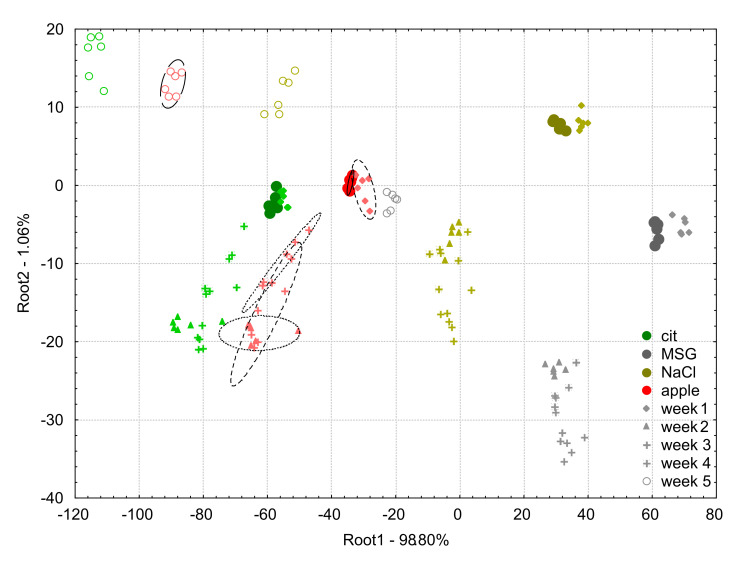
Linear discriminant analysis plots for raw data (non-drift corrected) of the e-tongue measurements of apple juice samples (red symbols) and model solutions: citric acid (green symbols), NaCl (gold symbols), and MSG (gray symbols). The solid circles indicate the measurements on week 0 which was used to build the linear discriminant analysis model, and the different symbols (diamond, triangle, plus, asterisk, and circle) stand for the different weeks 1–5, respectively.

Figure 5 shows the score plot of the LDA model built on the apple juice samples and the model solutions (0.01 M citric acid, MSG, NaCl) measured at week 0 and the projected data points of week 1, week 2, week 3, week 4, and week 5 of e-tongue measurements using the non-drift corrected data.

The average classification accuracies of the LDA classification for apple juice samples using the raw data (non-drift corrected) were 100%, 100%, 11.1%, 0%, 16.7%, and 0% for the samples measured in weeks 0 to 5, respectively. Considering the other solutions, on week 0, there was no misclassification observed.

For week 1, 22.2% of NaCl was misclassified as MSG, and 33.3% of citric acid to the group to apple juice. From week 2, the classification was completely unreliable and resulted in unacceptable misclassifications among all the four groups.

For week 2, 44.4% of MSG was misclassified as NaCl, 33.3% of NaCl as apple juice, 5.6% of citric acid as apple juice.

For week 3, 16.7% of MSG was classified as NaCl, 50% of NaCl as apple juice.

For week 4, 27.8% of MSG was classified as NaCl, and 38.9% of NaCl as apple juice.

For week 5, 100% of MSG was classified as apple juice, 27.8% of NaCl as apple juice.

#### 3.4.2. Additive Correction Relative to Whole Sample Set

**Figure 6:**
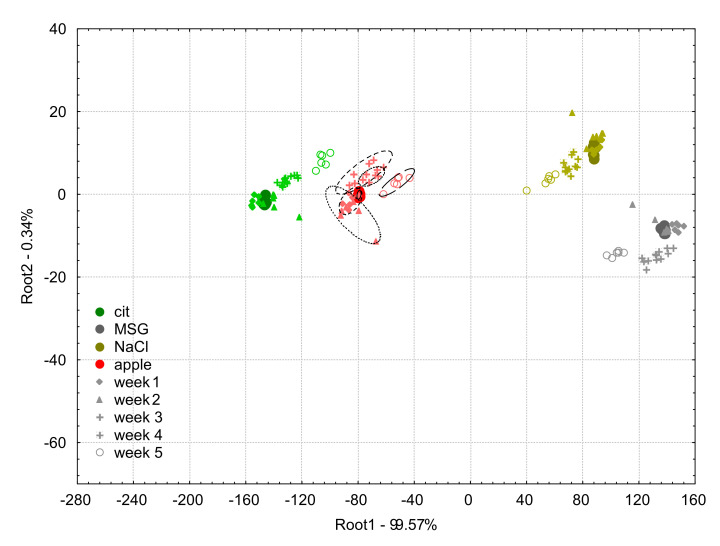
Linear discriminant analysis plots for the drift corrected dataset using the additive correction relative to the whole dataset method of the e-tongue measurements of apple juice samples (red symbols) and model solutions: citric acid (green symbols), NaCl (gold symbols), and MSG (gray symbols). The solid circles indicate the measurements on week 0 which was used to build the linear discriminant analysis model, and the different symbols (diamond, triangle, plus, asterisk, and circle) stand for the different weeks 1–5, respectively.

The LDA score plot of the model built on the apple juice samples and the model solutions (0.01 M citric acid, MSG, NaCl) measured at week 0 is presented in Figure 6 with the projected data points of week 1, week 2, week 3, week 4, and week 5 of e-tongue measurements after the application of the additive correction relative to the whole sample set method.

The classification accuracies of the LDA classification for apple juice samples of the drift corrected data using the additive correction relative to the whole dataset method were 100% for the samples measured in all the six weeks.

Considering the model solution samples, on week 0 and week 1, all samples were correctly classified.

On week 2 and 3, 5.6% of citric acid was misclassified to the group of apple juice samples.

On week 4, 27.8% of citric acid was misclassified to the group of apple juice samples.

On week 5, 66.7% of citric acid was classified as the apple juice sample.

#### 3.4.3. Additive Correction Relative to Reference Samples

**Figure 7:**
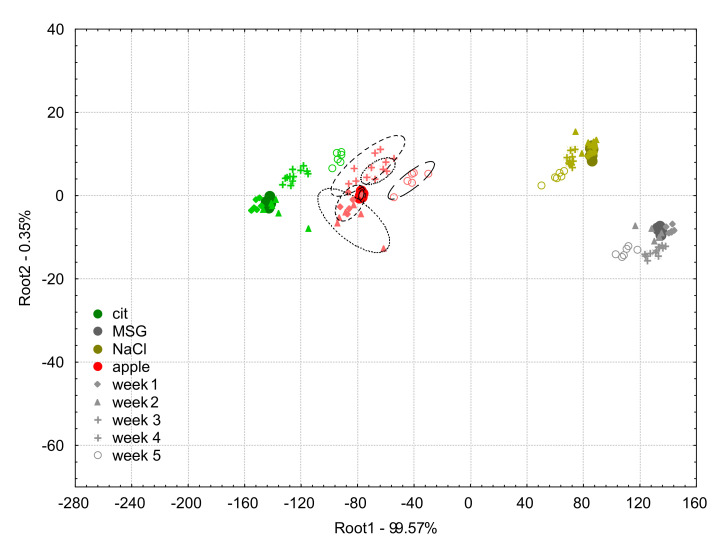
Linear discriminant analysis plots for the drift corrected dataset using the additive correction relative to reference samples method of the e-tongue measurements of apple juice samples (red symbols) and model solutions: citric acid (green symbols), NaCl (gold symbols), and MSG (gray symbols). The solid circles indicate the measurements on week 0 which was used to build the linear discriminant analysis model, and the different symbols (diamond, triangle, plus, asterisk, and circle) stand for the different weeks 1–5, respectively.

Figure 7 shows the LDA score plot of the classification model built on the apple juice samples and the model solutions (citric acid, MSG, NaCl) measured at week 0 and the results of the projection of the data points acquired between week 1, and week 5 with the e-tongue after applying the additive correction relative to the reference samples method.

The classification accuracies of the LDA classification for apple juice samples using the additive correction relative to the reference samples drift correction method were 100% for the samples measured on week 0, 1, 4, and 5, and 94.4% on week 2 and 3, respectively.

There were no misclassifications observed among the model solutions on week 0 and 1.

For week 2, 11.1% of citric acid was classified as apple juice, and 5.6% of apple juice as citric acid.

For week 3, 5.6% of citric acid was classified as apple juice, and 5.6% of apple juice as citric acid.

For week 4, 33.3% of citric acid was classified as apple juice.

For week 5, 66.7% of citric acid was classified as apple juice, and 5.6% of MSG as NaCl.

#### 3.4.4. Multi Sensor Linear Correction

**Figure 8:**
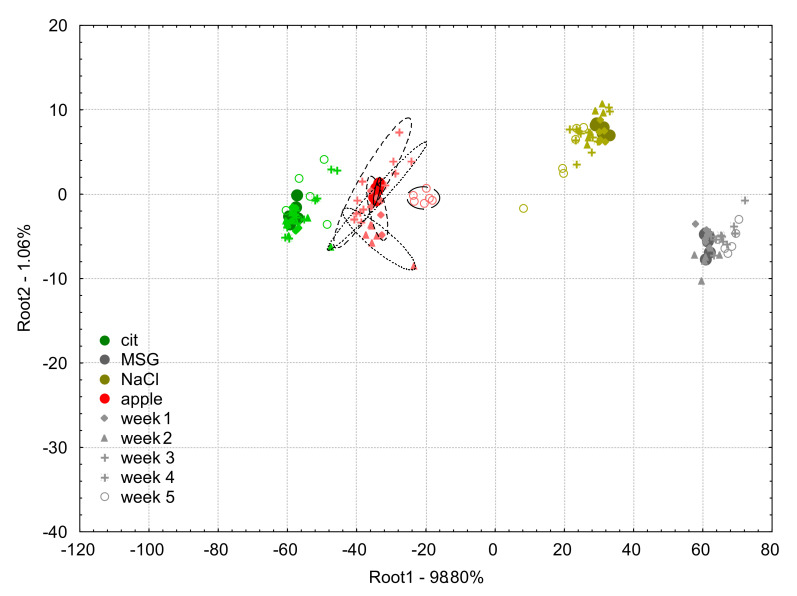
Linear discriminant analysis plots for the drift corrected dataset using the multi sensor linear correction method of the e-tongue measurements of apple juice samples (red symbols) and model solutions: citric acid (green symbols), NaCl (gold symbols), and MSG (gray symbols). The solid circles indicate the measurements on week 0 which was used to build the linear discriminant analysis model, and the different symbols (diamond, triangle, plus, asterisk, and circle) stand for the different weeks 1–5, respectively.

The LDA plot of apple juice measurement and the model solutions (citric acid, MSG, NaCl) built based on the data of week 0 is shown in Figure 8 presenting the results of the projections of the data of e-tongue experiments between week 1 and week 5 after the drift correction by the multi sensor linear correction.

The classification accuracies of the LDA classification for apple juice samples based on the drift corrected data using the multi sensor linear correction were 100% for the samples measured in week 0, 1 and, 5, and 93.3% and 94.4% in week 2 and 4, respectively.

There was no misclassification found on week 0 and week 1 among the model solutions.

For week 2, 11.1% of citric acid was classified as apple juice, 5.6% of NaCl as MSG, and 11.1% of apple juice as citric acid.

For week 3, 11.1% of citric acid was classified as apple juice.

For week 4, 5.6% of citric acid was classified as apple juice.

For week 5, 11.1% of citric acid was classified as apple juice.

#### 3.4.5. Component Correction

**Figure 9:**
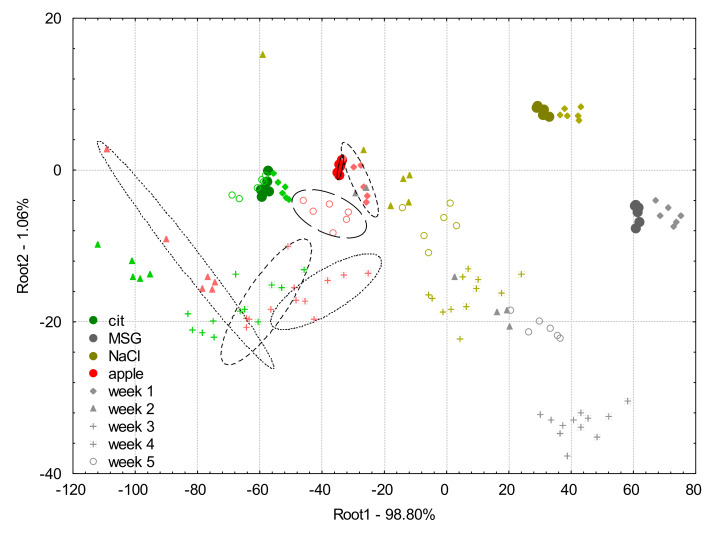
Linear discriminant analysis plots for the drift corrected dataset using the component correction method of the e-tongue measurements of apple juice samples (red symbols) and model solutions: citric acid (green symbols), NaCl (gold symbols), and MSG (gray symbols). The correction was performed based on pairing the first and last week. The solid circles indicate the measurements on week 0 which was used to build the linear discriminant analysis model, and the different symbols (diamond, triangle, plus, asterisk, and circle) stand for the different weeks 1–5, respectively.

The LDA plot of apple juice measurement and the model solutions (citric acid, MSG, NaCl) built based on the data of week 0 is shown in Figure 9, presenting the results of the projections of the data of e-tongue experiments between week 1 and week 5 after the drift correction by component correction based on pairing the first and last week.

**Figure 10:**
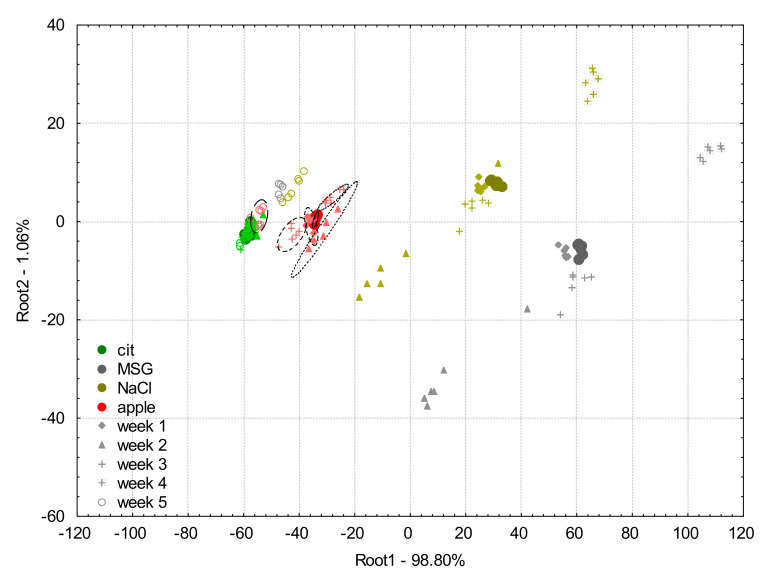
Linear discriminant analysis plots for the drift corrected dataset using the modified component correction method of the e-tongue measurements of apple juice samples (red symbols) and model solutions: citric acid (green symbols), NaCl (gold symbols), and MSG (gray symbols). The correction was performed based on pairing each week to week 0. The solid circles indicate the measurements on week 0 which was used to build the linear discriminant analysis model, and the different symbols (diamond, triangle, plus, asterisk, and circle) stand for the different weeks 1–5, respectively.

The LDA plot of apple juice measurement and the model solutions (citric acid, MSG, NaCl) built based on the data of week 0 is shown in Figure 10, presenting the results of the projections of the data of e-tongue experiments between week 1 and week 5 after the drift correction by component correction performed based on pairing each week to week 0.

The classification accuracies of the LDA classification for apple juice samples based on the drift corrected data using the multi sensor linear correction were 100% for the samples measured in week 0, 1, 2, and 4, and 83.3% and 0.0% in week 3 and 5, respectively.

There was no misclassification found on week 0 among the model solutions.

For week 1, 33.3% of MSG was classified as NaCl.

For week 2, 61.1% and 27.8% of MSG was classified as NaCl and apple juice, 50% of NaCl as apple juice.

For week 3, 16.7% of apple juice was classified as citric acid.

For week 4, 33.3% of NaCl was classified as MSG.

For week 5, 66.7.3% and 33.3% of MSG was classified as citric acid and apple juice, 44.4% and 55.6% of NaCl as citric acid and apple juice, 100.0% of apple juice as citric acid.

#### 3.4.6. Comparison of the Different Correction Method

**Figure 11:**
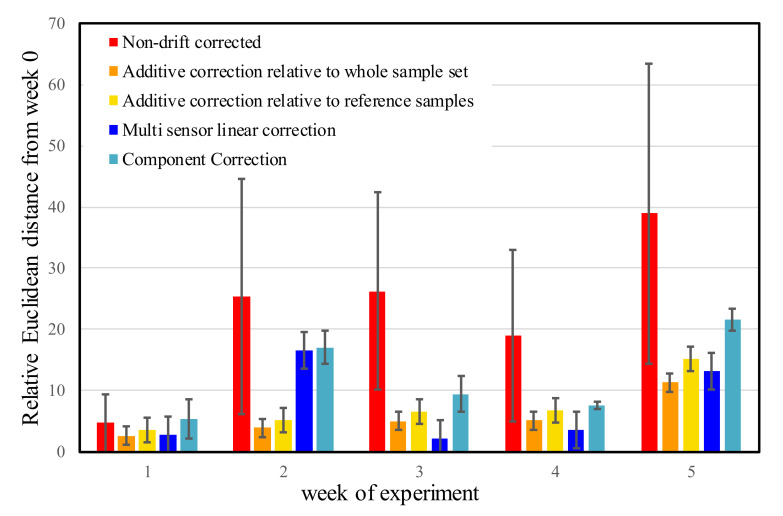
Comparison of the mean and standard deviation of the relative Euclidean distances of the apple juice samples from the center of week 0 calculated in the space of linear discriminant analysis models built based on the non-corrected and differently drift corrected data of e-tongue measurements. The intervals show the variation in distance for the threefold linear discriminant analysis cross-validation results.

Figure 11 shows the relative Euclidean distances from the center of the week 0 measurements for apple juice after one week, two weeks, three weeks, four weeks, and five weeks for the non-drift corrected and the for each developed drift correction method.

From Figure 11, it can be observed that except the first week, all the drift corrections drastically decreased the Euclidean distances of the apple juice samples from the center of the week 0 measurements. The distances do not show significant differences among the methods except for measurement done in week 2 where, the multi sensor linear correction and component correction provided significantly poorer correction. Finally, the corrections show similar effectiveness in all the weeks until week 4; on week 5, the effectiveness of the corrections were significantly worse.

## 4. Discussion

### 4.1. Effect of Temperature on the Sensor Signals

The results from Figure 2, demonstrate that temperature had a significant effect on the sensor signals. The apple juice samples having different temperatures were discriminated based on the first factor (Root 1), which contains about 98% of the variance between the groups. The apple juice samples of different concentrations measured at the same temperature were discriminated based on the second factor (Root 2), which contains only 2% of the variance between the groups. The relationship between the multidimensional distances of the sensor signals and the sample temperature could be approximated by a linear connection. The slope of the regression model assumes about 50-unit change for the multi-dimensional Euclidean distance in the seven-dimensional sensor space with a change of 1 °C of the apple juice sample. The change of apple juice concentration also caused a fairly linear change in the multi-dimensional Euclidean distance of the sensors, but with much lower effects on it. This caused approximately a seven unite change in the multi-dimensional Euclidean distance, with a change of 1% concentration of the apple juice sample.

### 4.2. Effect of Cross Contamination

Results of experiments performed to monitor the effect of cross-contamination showed a monotone increase in the pH of the apple juice which increased approximately by 0.1 for the repeat in the e-tongue measurement. This may be due to the distilled water being transmitted by the sensor head from the cleaning fluid to the apple juice samples. This phenomenon confirmed that cross contamination can influence e-tongue measurements especially in the case of measurement containing samples that are highly different in concentrations.

### 4.3. Analysis of Memory Effect

In Figure 4, the relative position of the sample groups on the PCA score plots was different from each other based on both PC1 and PC2. The standard deviation for the repeated measurements of the same sample were different for the different sample orders. These observations confirm that the sensor signals can be affected by the measurement order and can influence the time required to achieve equilibrium stage for the sensors. In addition, citric acid is chemically the closest in composition to apple juice and this could be why NaCl and MSG samples were grouped in the positive area of PC1 axis but citric acid and apple juice were grouped in the negative area of PC1 axis, regardless of measuring order. These could be attributed to the memory effect on e-tongue sensors.

### 4.4. Development and Comparison of the Different Drift Correction Methods

#### 4.4.1. Rawdata (Non-Drift Corrected)

From Figure 5, it can be observed that all the samples measured on week 0 had small inter group distances and they were well separated. That is why the threefold cross validation also resulted in 100% correct classification. Data points of all the four tested samples in the following weeks presented similar patterns in terms of variation, compared to their counterparts measured in week 0. For week 1, the groups were found relatively close to those of week 0 but became substantially higher for week 2. For week 3 and 4, the data points were projected by the model between groups in week 0 and week 2 showing the non-linear characteristic of the change in sensor signals. In spite of the less ideal condition on week 4 when cleaning fluids were not replaced in every round of experiments, no extreme variations were observed. Finally, on week 5, the data points of the measurements were projected substantially further from their week 0 locations and the direction was close to perpendicular to the direction observed for the previous weeks. The reason could be because the temperature during the measurement was different. The inter group distances increased with the respective longer experimental periods (weeks), except the last week where the inter group distances were similar to week 1.

#### 4.4.2. Additive Correction Relative to Whole Sample Set

From Figure 6, it can be observed that the correction led to significant improvements in the results. The data points of each of the tested samples presented more compact groups with low intergroup distance, compared to the non-corrected ones. After threefold cross-validation, apple juice samples were corrected with an accuracy of 100% throughout the entire six-week period of the experiment. For week 5, the sample groups showed only clear separation from the groups of the previous weeks for all the tested samples, but it did not cause any misclassifications for the apple juice measurements. The additive correction based on the whole sample set can therefore be said to be effective for the correction of drift occurring within six weeks of experimental period.

#### 4.4.3. Additive Correction Relative to Reference Samples

From Figure 7, the effectiveness of the additive correction relative to reference samples method was similar to the additive correction relative to whole sample set one. It can be noticed, however, that some of the data points of week 2 and week 3 showed higher distance from their week 0 groups, which was also characterized by the slightly lower correct classification results. The biggest variation was observed in the data of week 5, which suggests that effects of change in the sample temperature may not be fully corrected by this method. The additive correction relative to reference samples method can therefore be said to be effective for correcting drift occurring within only controlled environments, variations in temperature can be a challenge for this approach.

#### 4.4.4. Multi Sensor Linear Correction

The results of multi sensor linear correction presented Figure 8 showed similar effectiveness of this correction method in comparison to the additive correction methods. There were no misclassifications for week 1, week 2, and week 5, but weaker results were found for the apple juice classifications for week 2 and 4. Data points of week 5 perfectly overlapped with the groups of the model samples for the three sample solutions but showed some separation for the data points of apple juice samples. This implies different change in the sensor signals by the different type of samples. The multi sensor linear correction can therefore be said to be effective for correcting drift in e-tongue measurements with relatively high efficiency for long-term experimental periods of up to six weeks using the samples under study.

#### 4.4.5. Component Correction

Comparing Figure 9 and Figure 10, the modified component correction method showed better results than the component correction method reported by Artursson et al. [28]. The component correction method was only effective in correcting drift for week 0 and week 2 of experiments, but the modified component correction method could correct drift for up to four weeks experiment for MSG and NaCl and even in all six weeks of experiment for citric acid and apple juice.

### 4.5. Comparison of the Different Drift Correction Methods

All drift corrections provided apparent improvement for all the samples and LDA plots showed similar patterns for all the different drift corrections. The results of NaCl and MSG showed significant improvement compared to those of citric acid and apple juice, although their raw data showed higher separations. From the LDA plots, none of the corrections resulted in a perfect overlap for the different weeks, but they all provided high classification accuracy for apple juice samples. The component correction by Artursson et al. [28] was effective in correcting drift for short experiments, but the modified version proved to be capable of correcting drift in longer term experiments of up to six weeks. The component correction method had the highest mean and standard deviation in the relative Euclidean distances of the apple juice samples when all the drift correction methods were compared in Figure 11. This suggests that, comparatively, it was the least effective in correcting drift in datasets from long-term experiments. The modified version of this however, showed contrary results that were effective in correcting for up to six weeks of experiments for citric acid and apple juice.

The multi sensor linear correction did not show better performance than the mathematically simpler additive corrections: the additive corrections were characterized by lower inner group distances for the weekly experiments compared to the other correction methods.

The reason for that could be because the additive corrections were applied for every single sample repetition but the multi sensor linear correction was applied on the whole group of the given sample for the different weeks. Supposedly, the combination of these two methods could provide slightly better results, but from all the methods compared in this study, the additive correction based on the whole sample set gave the best results: it was capable of correcting drift occurring within six weeks of experiments with 100% classification accuracy.

## 5. Conclusions

Literature has shown that temperature, memory effect, and cross-contamination can lead to drift in e-tongue sensors. This was tested in this paper by performing an experiment for five weeks to assess the impact of these factors on e-tongue sensors and develop drift correction methods.

From the results, the relationship between the sensor signal and the sample temperature could be approximated by linear connection after temperature was shown to influence e-tongue results. The pH of the apple juice increased approximately by 0.1 in the experiment, which was suspected to be caused by the distilled water through the sensor head.

The measurement of the apple juice samples and model solutions applying different sample order resulted in significant differences based on the Euclidian distances between the sample groups and scatter caused by the memory effect on e-tongue sensors. This could be attributed to the altered sensor stabilization and sensitivity.

Linear discriminant analysis of the non-drift corrected data from the e-tongue confirmed the influence of temperature, memory effect, and cross-contamination on sensor signals, so four different drift correction methods were developed and applied: multi sensor linear correction, additive correction based relative to the whole data set, the additive correction relative to the reference samples method, and modified component correction.

Linear discriminant analysis predictions could not entirely correct the data to provide perfect classifications but the results confirmed that the applied drift correction methods significantly improved the long-term measurement results of the electronic tongue and could be adapted for industrial purposes. The additive correction based on the whole sample set could correct drift occurring within six weeks of the experiment and was as such found to be the most effective drift correction method.

## Figures and Tables

**Table 1:** Sample preparation and experimental conditions for drift correction measurements.

Week	Experimental Samples	Experimental Conditions	
		Temperature	Cleaning Solution
0	10 times dilution of 100% fresh apple juice and model solutions: Citric acid, MSG and NaCl	25 °C ± 0.2 °C (controlled)	Replaced between each sample repeat
1	10 times dilution of 100% fresh apple juice and model solutions: Citric acid, MSG and NaCl	25 °C ± 0.2 °C (controlled)	Replaced between each sample repeat
2	10 times dilution of 100% fresh apple juice and model solutions: Citric acid, MSG and NaCl	25 °C ± 0.2 °C (controlled)	Replaced between each sample repeat
3	10 times dilution of 100% fresh apple juice and model solutions: Citric acid, MSG and NaCl	25 °C ± 0.2 °C (controlled)	Replaced between each sample repeat
4	10 times dilution of 100% fresh apple juice and model solutions: Citric acid, MSG and NaCl	25 °C ± 0.2 °C (controlled)	No replacement
5	10 times dilution of 100% fresh apple juice and model solutions: Citric acid, MSG and NaCl	23 °C (uncontrolled)	No replacement
